# *Intra-vital* imaging of mesenchymal stromal cell kinetics in the pulmonary vasculature during infection

**DOI:** 10.1038/s41598-021-83894-7

**Published:** 2021-03-04

**Authors:** Claire H. Masterson, Arata Tabuchi, Grace Hogan, Glenn Fitzpatrick, Steven W. Kerrigan, Mirjana Jerkic, Wolfgang M. Kuebler, John G. Laffey, Gerard F. Curley

**Affiliations:** 1 Department of Anesthesia, Keenan Research Centre for Biomedical Science of St Michael’s Hospital, St. Michael’sHospital, 30 Bond Street, Toronto, Canada; 2 Department of Critical Care Medicine, Keenan Research Centre for Biomedical Science of St Michael’s Hospital, St. Michael’s Hospital, 30 Bond Street, Toronto, Canada; 3 Department of Surgery, Keenan Research Centre for Biomedical Science of St Michael’s Hospital, St. Michael’sHospital, 30 Bond Street, Toronto, Canada; 4 Regenerative Medicine Institute (REMEDI) at CÚRAM Centre for Research in Medical Devices, Biomedical Sciences Building, National University of Ireland Galway, Galway, Ireland; 5 Department of Anesthesia, University of Toronto, Toronto, Canada; 6 Department of Physiology, University of Toronto, Toronto, Canada; 7 Interdepartmental Division of Critical Care, University of Toronto, Toronto, Canada; 8 Department of Anaesthesia, National University of Ireland, Galway, Ireland; 9 Institute of Physiology, Charité-Universitätsmedizin Berlin, Berlin, Germany; 10 Department of Anaesthesia and Critical Care, Royal College of Surgeons in Ireland, Dublin, Ireland

**Keywords:** Mesenchymal stem cells, Respiratory distress syndrome, Preclinical research, Infection, Acute inflammation, Sepsis

## Abstract

Mesenchymal stem/stromal cells (MSCs) have demonstrated efficacy in pre-clinical models of inflammation and tissue injury, including in models of lung injury and infection. Rolling, adhesion and transmigration of MSCs appears to play a role during MSC kinetics in the systemic vasculature. However, a large proportion of MSCs become entrapped within the lungs after intravenous administration, while the initial kinetics and the site of arrest of MSCs in the pulmonary vasculature are unknown. We examined the kinetics of intravascularly administered MSCs in the pulmonary vasculature using a microfluidic system in vitro and intra-vital microscopy of intact mouse lung. In vitro*,* MSCs bound to endothelium under static conditions but not under laminar flow. VCAM-1 antibodies did not affect MSC binding. *Intravital* microscopy demonstrated MSC arrest at pulmonary micro-vessel bifurcations due to size obstruction. Retention of MSCs in the pulmonary microvasculature was increased in *Escherichia coli*-infected animals. Trapped MSCs deformed over time and appeared to release microvesicles. Labelled MSCs retained therapeutic efficacy against pneumonia. Our results suggest that MSCs are physically obstructed in pulmonary vasculature and do not display properties of rolling/adhesion, while retention of MSCs in the infected lung may require receptor interaction.

## Introduction

Mesenchymal stem/stromal cells (MSCs) have demonstrated efficacy in pre-clinical models of inflammation and tissue injury, including in models of lung injury and infection^1–4^, and in systemic sepsis^5–7^. A large proportion of MSCs become entrapped within the lungs after intravenous administration^8–11^. Similar to the multistep paradigm of leukocyte adherence^12^, in vitro and *intra-vital* studies have demonstrated that MSCs tether, roll, firmly adhere and transmigrate upon contact with the endothelium in the systemic vasculature^13^, particularly in capillaries and venules^14^. Furthermore, cellular adhesion molecules associated with leukocyte and platelet adhesion have been reported to play a key role in mediating MSC adhesion and transmigration^13,15^. MSCs have been reported to home to injured areas in the systemic vasculature such as the heart, liver and kidney^16–18^ via specific adhesion receptors and strategies to increase MSC migration to these organs has led to improved outcomes in preclinical studies^19,20^.

The initial kinetics and the site of arrest of MSCs in the pulmonary vasculature are currently unknown. There are several reasons why MSC trafficking in the lung may depend on other factors besides MSC-endothelial interactions. First, leukocyte trafficking in the pulmonary microvasculature is unique and differs in important ways from other, non- pulmonary vascular beds^21,22^: the dense capillary network of the lung serves as a major site of physiological sequestration of leukocytes (margination)^23^; the capillary bed is the major site of leukocyte retention and extravasation in the lung^24^ (as opposed to post- capillary venules in the systemic circulation); changes in leukocyte rigidity during inflammation ‘trap’ the cell within the pulmonary capillaries^25^, while adhesion molecules appear to have little impact on initial leukocyte sequestration within the lungs^26,27^. Moreover, the size and rigidity of cells impacts on their sequestration, such that MSCs may become mechanically trapped in smaller size arterioles and capillaries instead of, or in addition to, receptor mediated interactions. The influence of inflammatory mediators, such as those present in bacterial infection, influence the expression of endothelial receptors impacting receptor-mediated binding and extravasation. As described by Ince and colleagues^28^ the pathogenic effects of sepsis in the endothelium involves glycocalyx destruction leading to integrin and selectin exposure, increasing trapping and transmigration of leukocytes, which could potentially impact MSC adhesion and retention in the lung during pneumonia.

The precise fate of MSCs after sequestration in the lung has also not been clearly defined. Persistence of MSCs in the lung 24 h after systemic administration is low^8,9,29^. This may reflect passage onwards through the lungs^29^ and on to distal organs or cell death due to immune recognition^9^. In addition, there is emerging evidence that MSCs can extravasate from the systemic vasculature in a murine model of dermal inflammation^14^, and from the pulmonary vasculature into the alveolus during *Escherichia coli* pneumonia^4^.

We used an established model of *intravital* microscopy of intact mouse lung to address these knowledge gaps, which allowed for real time imaging of MSCs during and after intravenous administration^30^. We hypothesized that MSCs are physically retained in the pulmonary vasculature, and that persistence of these cells is similar in healthy animals and in animals with *E coli* pneumonia.

## Results

### MSC adhesion to pulmonary microvascular endothelium under static conditions in vitro

**Figure 1:**
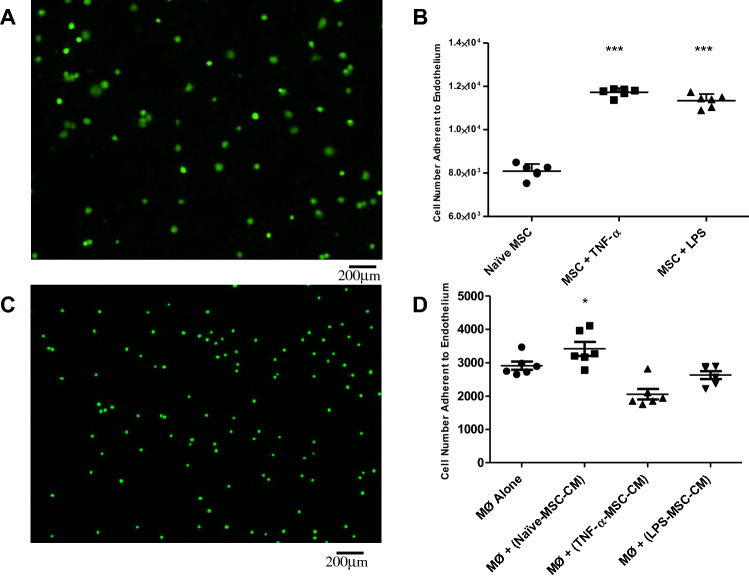
In situ adhesion assays were performed using fluorescently labelled MSCs or monocytes (Mo) added to TNF-α-activated monolayers of hPMVECs. Pre-activation of MSCs with TNF-α or LPS enhanced MSC adhesion ability (**A**,**B**). Treatment of U937 monocytes with conditioned media from Naïve MSCs allowed for greater adhesion, however conditioned media from MSCs pre-treated with inflammatory cytokines did not enhance this effect (**C**,**D**). MSC: Mesenchymal Stem Cell; TNF-α: Tumour Necrosis Factor α; hPMVEC: human pulmonary microvascular endothelial cells; LPS: Lipopolysaccharide; Mo: Monocyte; CM: Conditioned Media. Columns represent mean + SD (n = 6), * = *P* < 0.05 versus Control.

Naïve and pre-activated MSCs (Fig. 1A,B) or U937 monocytes exposed to naïve and pre- activated MSC-CM (Fig. 1C,D) were added to confluent monolayers of hPMVECs primed with TNF-*a*. By imaging fluorescent MSCs bound to the endothelial cell monolayer 1 h after addition, we confirmed previous observations that MSCs bind to endothelial cells under static conditions, and pre-activation of stromal cells enhances this monocyte/MSC-endothelial interaction. (Fig. 1A,B). The addition of MSC-CM from naïve MSCs increased the binding capacity of U937 monocytes but interestingly the addition of MSC-CM from pre-activated MSCs did not enhance the binding capacity of monocytes (Fig. 1C,D). ICAM is a major cell wall adhesion receptor involved in many cell–cell interactions at the interface of the vascular endothelium^21^. Preincubating the hPMVECs with an antibody against ICAM for 2 h before addition of the MSCs failed to have any significant effect on binding (Supplemental Figure 1).

### MSC adhesion to pulmonary microvascular endothelium under shear stress in vitro

**Figure 2:**
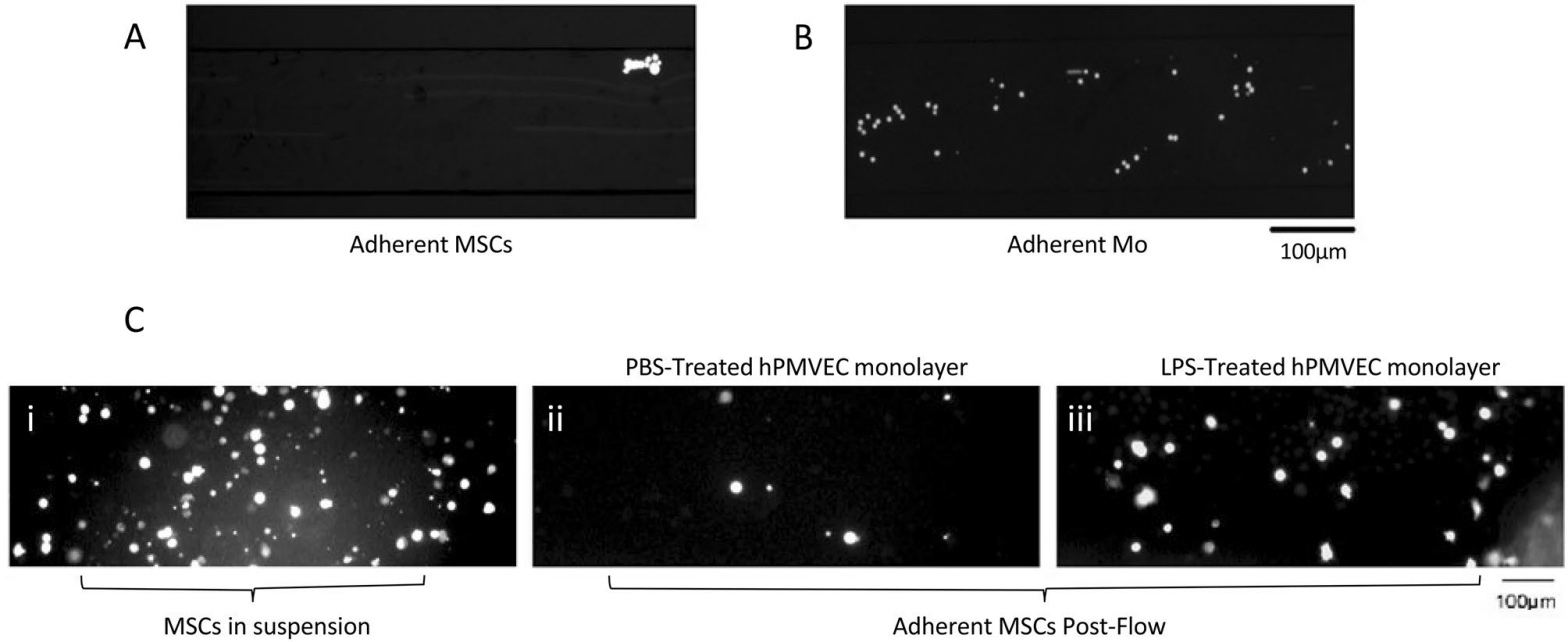
hPMVECs were seeded in BioFlux microfluidics channels to form monolayers and the application of 2 dyn/cm^2^ pressure allowed fluorescently labelled MSCs to pass over the monolayer under shear flow. Still images from recordings indicate little to no binding of MSCs (**A**) compared to Mo control cells under the same conditions (**B**). hPMVEC monolayers were treated with PBS (**C**, Panel ii) or LPS (**C**, Panel iii) before MSC suspensions were passed over the layers. Flow was stopped and the cells were allowed to settle on the monolayer for 30minutes (**C**, Panel i) before re-initiation of the flow. Once all cells had passed through the chamber, images were acquired of the remaining, firmly adherent cells (**C**, Panels i, ii). MSC: Mesenchymal Stem Cell; LPS: Lipopolysaccharide; Mo: Monocyte; hPMVEC: human pulmonary microvascular endothelial cells.

We used a microfluidic system to mimic shear-flow conditions encountered in the pulmonary vasculature. hPMVECs monolayers were grown in collagen-coated BioFlux microfluidic channels and incubated with TNF-*a* for 4 h as described (Supplemental Figure 2A)^31^. Over 4 min of MSCs flowing through the microfluidic channels there was limited interaction of MSCs with the endothelium (Fig. 2A and Supplemental Video 1). MSCs did not exhibit rolling behavior on endothelium and no single MSCs were seen to adhere to the endothelial layer (Supplemental Figure 2B and Supplemental Video 1), even after pre-treating MSCs with TNF-*a* or LPS (Supplemental Figure 2C,D). This assay was repeated with and without TNF-*a* stimulation of hPMVECs, and with human blood outgrowth endothelial cells (BOECs). Again, little to no MSCs were seen to interact with the endothelium. Adjusting the flow rate up to 10 dynes/cm^2^ or down to 0.5 dynes/cm^2^ still did not lead to MSC rolling or adherence to the endothelium following TNF-*a* or LPS pre-activation. In contrast, monocytes displayed marked rolling behavior and firm adhesion to PMVECs in the microfluidics chamber at a wall stress of 2 dyne/cm^2^ (Fig. 2B, Supplemental Video 2). A further experiment was conducted to assess MSC adhesion to the hPMVEC monolayer after an initial period of stasis. MSCs were added to the channels and allowed to adhere for 30 min following which a shear stress of 2 dyne/cm^2^ was applied. Following initial stasis, MSCs were found to adhere to PBS-treated endothelial layers (Fig. 2C, Panel ii) and more so to LPS-activated endothelial monolayers (Fig. 2C, panel iii).

### First Pass kinetics of MSCs in intact mouse lung

**Figure 3:**
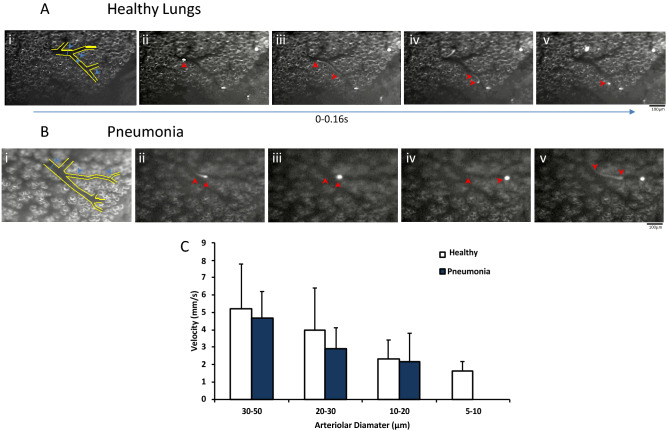
Real-time Intra-vital recording of MSC accumulation in the murine lung microvasculature allowed for measurements of velocity in healthy (**A**) and *E. coli* infected lung (**B**). Velocities of individual MSCs (movement indicated by red arrows, A, B, Panels ii–v) were measured in different pulmonary vessel diameters (Blue arrows, **A**,**B**, Panel i) Quantitative analysis showed that there was no significant differences in MSC velocities in different vessel diameters between healthy and pneumonia mouse models (**C**). MSC: Mesenchymal Stem Cell; Columns represent mean + SD (n = 4–13 from 6 separate animals).

We next examined the first pass kinetics of MSCs in the intact mouse lung of either healthy animals or in animals with moderate *E. coli* pneumonia. We employed intravital fluorescence video-microscopy to directly visualize the behavior of fluorescently—labeled MSCs in the different diameter vessels of the pulmonary microvasculature (Fig. 3A,B; Panel i, blue arrows). Following their infusion, CFSE-MSCs almost immediately appeared in the microscopic field (Fig. 3A,B, panels ii–v, and Supplemental Videos 3 & 4). Analysis of the recordings of MSC passage through the pulmonary microcirculation, frame by frame (25FPS) (Fig. 3A,B; panels ii–v), showed that few of the cells passed directly through the microscopic field without stopping (Supplemental Videos 3 & 4). Similar to neutrophils, MSCs moved in hops through capillary segments (Supplemental Videos 3 & 4), eventually firmly adhering to the endothelium. MSCs were measured ex vivo to determine a cell to vessel diameter difference which would account for the halting of cell transit through the small pulmonary vessels (Supplemental Figure 3). Murine MSCs were significantly larger than mouse white blood cells. Measurement of MSC velocity prior to arrest was carried out according to vessel type and diameter (Fig. 3A,B (red arrows indicating movement of one cell) & C). The velocities of visualized MSCs was not consistent with the velocity of rolling cells (approx. 5 µm/s), but rather reflected the characteristic blood flow velocity profile RBCs in the pulmonary arteriolar vessel tree (Fig. 3C)^32, 33^.

**Figure 4:**
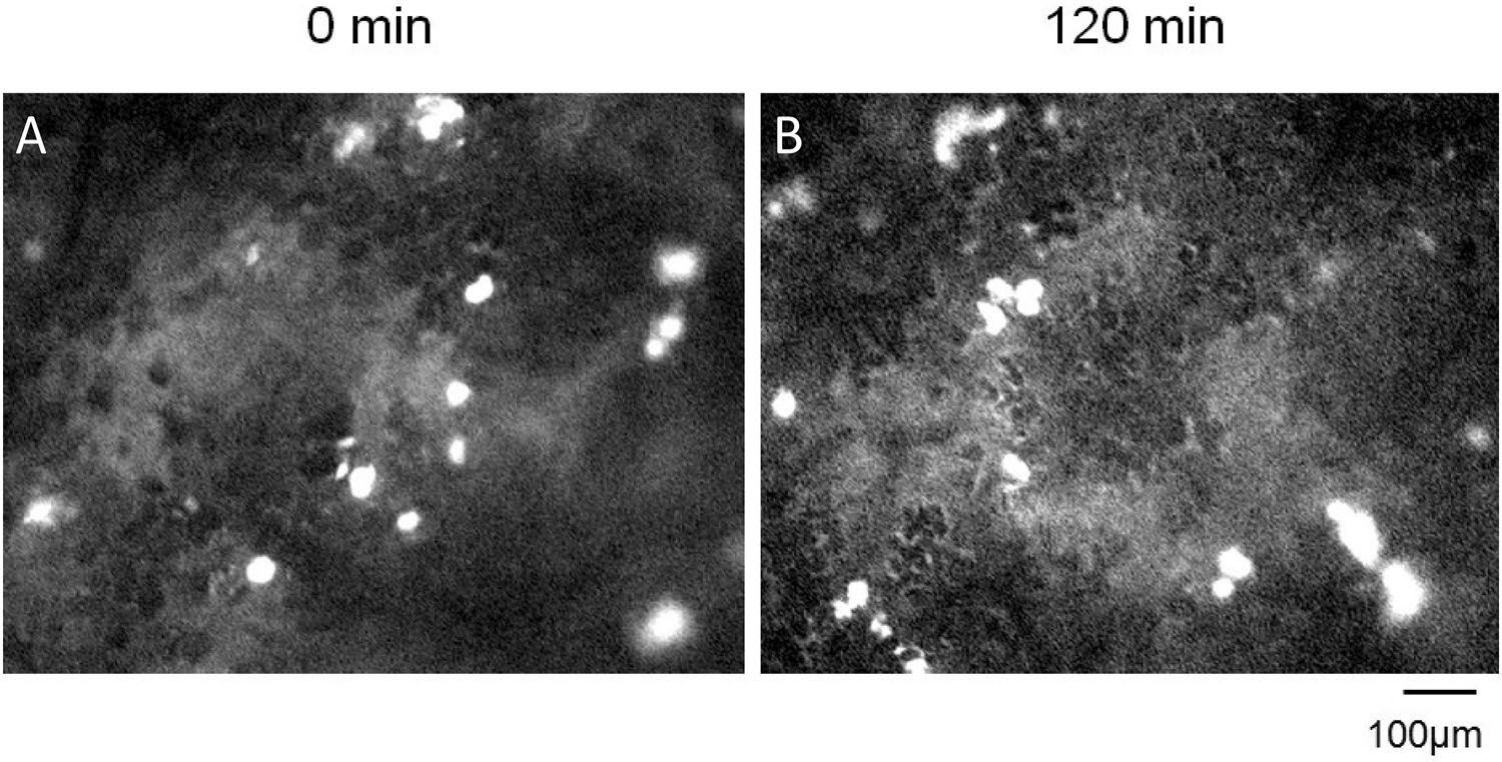
Administration of a FITC-labelled Dextran solution demonstrated the position of trapped MSCs in the pulmonary vasculature. Detection of FITC-labelled Dextran almost immediately after administration indicated that the MSCs were located in the post-arterial but not pre-venular capillaries (**A**). Cell position did not change 120 min after their infusion (**B**). FITC: Fluorescein isothiocyanate; MSC: Mesenchymal Stem Cell; *Note*: Images taken from different animals at each time-point.

Infusion of FITC-labelled Dextran was also utilized to determine the position of the trapped MSCs in the lung vasculature as previously described^34^. Dextran was visualized almost instantaneously following infusion, highlighting the arterioles in which the MSCs were lodged (Fig. 4A, Supplemental Video 5). After 120 min of MSC observation in vivo, separate animals were infused with FITC-Dextran to ascertain if MSC position had shifted. Analysis revealed that the MSCs had not moved to the pre-venular capillaries as indicated by the position of the MSCs distal to the dextran solution during initial arterial flooding (Fig. 4B).

### MSC retention in the pulmonary vasculature is increased by tissue injury

**Figure 5:**
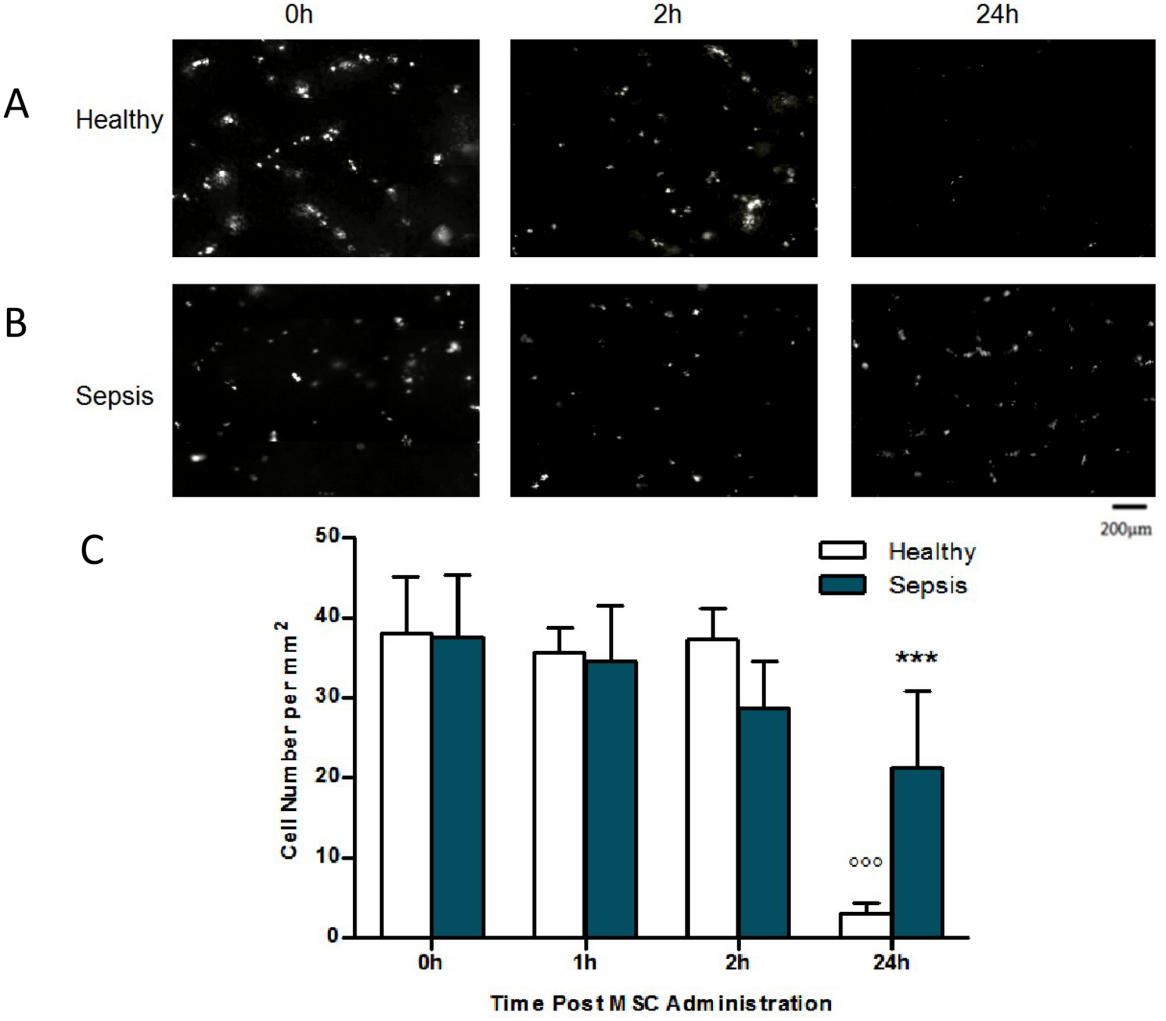
IVM was performed at 0 h, 2 h and 24 h post fluorescent MSC administration and composite images rendered from lung surface recordings (**A**,**B**). Quantitative analysis of cell numbers in composite images demonstrated no differences in cell retention between healthy and septic animals from 0 to 6 h (**C**). However, a significantly higher number of cells remained in animals with *E. coli* pneumonia 24 h post cell administration (**A**,**B**,**C**). IVM: Intravital Microscopy; MSC: Mesenchymal Stem Cell; Columns represent mean + SD (n = 2–5), * = *P* < 0.05 versus 24 h Healthy, ^o^ = *P* < 0.05 versus 0 h Healthy.

Following examination of in vivo MSC kinetics after intravenous infusion, we next compared the retention times for these cells in the lung vasculature in healthy versus *E. coli* pneumonia mice. Following a 4 h sham or *E. coli* pneumonia injury, mice received an intravenous infusion of CFSE-labelled MSCs as described and intravital microscopy was used to assess MSC retention at different time-points. Animals were examined at 0 h, 2 h, and 24 h post MSC infusion and composite images rendered from lung surface recordings (Fig. 5A,B). Quantitative analysis of cell numbers in composite images demonstrated no differences in cell retention in healthy versus *E. coli* pneumonia animals from 0 to 2 h, however, a significantly higher number of cells remained in animals with *E. coli* pneumonia 24 h post cell administration compared to healthy animals at this timepoint (Fig. 5C).

### MSCs in the lung vasculature demonstrate notable changes in cell morphology

**Figure 6:**
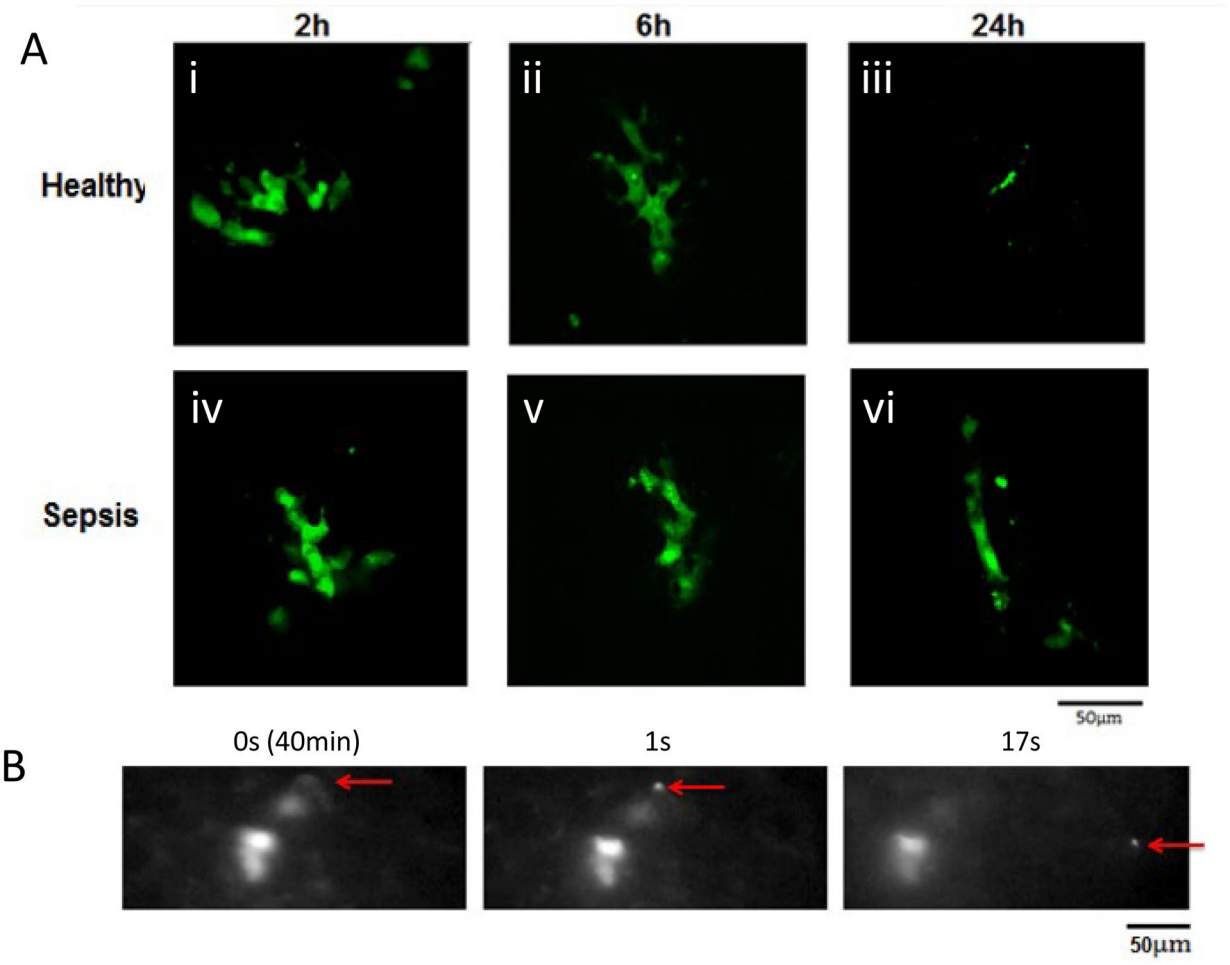
In vivo confocal microscopy allowed detailed analysis of cell structure at each time- point in healthy and pneumonia lungs with cell size differences discernibly evident at 24 h between MSCs in healthy and pneumonia lungs. The formation of micro-particles from the cells was indicated in post mortem confocal microscopy (**A**) and performing IVM using a 40X objective demonstrated the time-lapse release of fluorescent micro-particles (B, Red Arrow) approximately 40 min post cell administration IV (**B** i-iii) IVM: Intravital Microscopy; MSC: Mesenchymal Stem Cell.

Recordings of murine lung vasculature at different time-points post MSC administration indicate that the cells deformed over time to adapt to the shape of the vessel in which they were lodged (Supplemental Figure 4). Using fluorescent confocal intravital microscopy, we observed that the MSCs became highly distorted very soon after infusion of the cells with the appearance of smaller vesicles containing CFSE label. A similar degree of cell deformation was observed in both healthy and *E. coli* pneumonia mice (Fig. 6A, Panels i–vi) with the almost complete depletion of cells in healthy animals at 24 h (Fig. 6A Panel iii).

When observing cell deformation over time the release of several micro-vesicles measuring approximately 1 µm in diameter was captured (Fig. 6B, red arrow). The production of these microvesicles was detected 40 to 60 min post MSC administration to healthy and pneumonia animals in the majority of cells observed for this time-period (Fig. 6B, Supplemental Figure 5). Prolonged observation for quantification of vesicle release was impeded by the loss of fluorescent signal due to exposure.

### Fluorescently labelled MSCs retain their therapeutic effects in a mouse model of E. coli pneumonia

**Figure 7:**
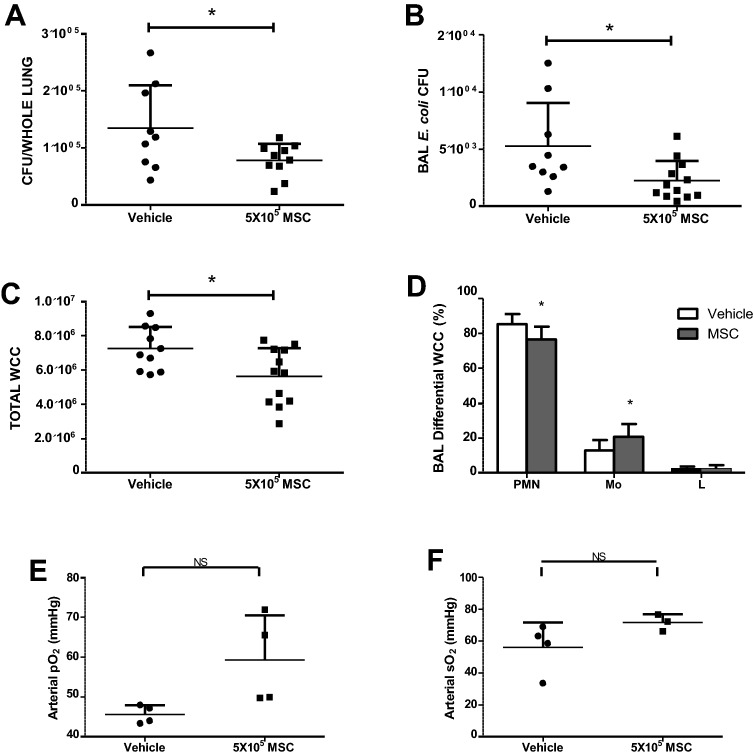
Administration of CFSE-labelled MSCs to murine models of E. coli pneumonia demonstrated that their therapeutic abilities were retained. Bacterial CFU were significantly reduced in BAL and lung tissue (**A**,**B**), total white cells counts and the fraction of inflammatory cells in the BAL were significantly reduced (**C**,**D**) and administration of MSCs resulted in a trend toward improved oxygenation (**E**,**F**).CFSE: Carboxyfluorescein succinimidyl ester; MSC: Mesenchymal Stem Cell; BAL: Bronchioalveolar lavage; CFU: Colony forming units. Columns represent mean + SD (n = 3–6),* = *P* < 0.05 versus Vehicle Control.

CFSE-labelled MSCs (or vehicle control) were administered intravenously to mice 4 h after intratracheal *E. coli* instillation. 44 h later animals were examined for MSC efficacy against *E. coli* infection. As previously demonstrated by our group and others^1^, MSCs significantly reduced the colony forming units of *E. coli* in both the BAL and lung tissue (Fig. 7A,B).

The total white cells count from the BAL indicated that administration of MSCs significantly reduced this and differential staining demonstrated that of these white cells the fraction of inflammatory neutrophils was significantly reduced, and the fraction of macrophages/monocytes was increased compared to vehicle control (Fig. 7C,D). Blood gas analyses revealed that the administration of MSCs resulted in improved arterial and saturated oxygen levels (Fig. 7E,F).

## Discussion

The current studies provide important insights into initial kinetics and site of arrest of systemically administered MSCs within the pulmonary microcirculation, and mechanisms mediating this interaction between the MSC and the pulmonary endothelium. Our finding that systemically administered MSCs become physically trapped in the arteriolar pulmonary microcirculation is novel, and contrasts with previously described MSC-endothelial interactions in the systemic circulation. The demonstration that lung injury increases the retention of physically entrapped MSCs in the pulmonary microcirculation, and that entrapped MSCs release microvesicles may provide targets to enhance the efficacy of MSC therapies in the injured lung.

Previous studies have suggested that MSCs adhere to the endothelium of systemic blood vessels via integrins^35^ and VCAM-1^13,15,36^. As the principal site of leukocyte adhesion and migration in the lung is the capillary bed^21^—a concept that was subsequently confirmed for MSC sequestration in the lung by our intravital microscopic studies (Fig. 3, 5, supplemental video 2)—we used primary human pulmonary microvascular endothelial cells (hPMVEC) in our in vitro experiments. Previous studies have not evaluated MSC rolling and adhesion on pulmonary microvascular endothelial cells under conditions of shear flow. One study has shown rolling of MSCs on human umbilical vein endothelial cells in vitro^13^; however, rolling velocities were high, at shear stress of up to 4.0 dynes/cm^2^, far higher than those typically encountered by rolling leukocytes^37^. To investigate the potential for MSCs to bind to endothelium, we developed in vitro models of ‘‘quiescent’’ and ‘‘activated’’ pulmonary microvascular endothelium. It is well documented that activation of the endothelium in response to injury or infection leads to the upregulation of adhesion molecules and chemokines that localize the recruitment of blood leukocytes to specific tissues and organs^38,39^. Endothelial cell activation with the inflammatory cytokine TNF-α is known to upregulate expression of adhesion molecules such as VCAM-1 and ICAM-1^40^ and for the purpose of our studies we used this to polarize our endothelial layers as standard. LPS was used as our in vitro endotoxin injury (where live Gram-negative bacteria use was not feasible) and has been shown to directly increase E-selectin and integrin counter receptor expression^41^. The absence of substantial MSC binding to the pulmonary endothelial monolayer corroborates with other studies which have shown a lack of rolling of MSCs on stimulated and unstimulated human and murine endothelial cells in vitro under flow conditions^42,43^.

The unique structure of the pulmonary microvasculature plays a major role in the transit and sequestration of large cells such as leukocytes in the lung. Spherical neutrophils, which are 6–8 µm in diameter, have prolonged transit times through pulmonary capillary segments that measure 2–15 µm (26 s versus 1.4–4.2 for RBCs)^21^. Interestingly, the neutrophils do not simply move slowly, but rather move in ‘hops’^44,45^. Measurement of hMSC dimension in suspension demonstrated diameters of 20–50 µm and predicted that obstruction would play a major role in sequestration in the pulmonary vasculature. Murine MSCs displayed slightly smaller diameters of 10–30 µm. Taken together, these data suggest that MSCs trapping at the end of the arteriolar vessel tree appears due to the structural characteristics of the vessels and the mechanical properties of MSCs which are unable to deform. MSC kinetics in mouse lung is consistent with impedance, and not with rolling or adhesion evident from the visualisation of an abrupt stop of the cells at vessel junctions, rather than a slowing along the length of the vessel due to rolling and adhesion.

It is a formidable concern that intravascular administration of MSCs pose a danger of vessel obstruction with subsequent ischemia or embolism in the pulmonary vasculature^46^. Here we demonstrate that despite the apparent obstruction of the vessels by the large MSCs, there was no apparent occlusion of the vessel as demonstrated by the free flow of dextran through the observed regions around the obstructed vessels and no gross appearance of embolism even 24 h post MSC administration. In fact, the break-down of the MSCs began approximately two hours post administration with an almost complete ablation of MSCs by 24 h in healthy models. This was attenuated in our murine sepsis model where cells were retained, mostly intact, in the pulmonary vasculature. It is well known that endothelial cells respond to infection by upregulating surface adhesion molecules luminally which facilitates the rolling and adhesion of leukocytes and ultimate extravasation to the infected tissues^47^. We hypothesise that the upregulation of these adhesion molecules, while they did not facilitate MSC rolling, played a role in the retention of the MSC at the site of rest in the vasculature.

Taken together the data generated in the in vitro and in vivo experiments demonstrate that the cells are obstructed rather than bound by endothelial adhesion molecules. When MSCs are allowed to sit static on endotoxin-treated endothelial layers in vitro or retained by size obstruction in the lumen of infected vessels that they are retained for prolonged periods of time. This implies a weak interaction with the endothelial adhesion markers which is strengthened over time resulting in an increase in the longevity of intact MSCs in injured lungs.

Our study demonstrates that there is no evidence to support the hypothesis that adhesion molecules mediate MSC transport/adhesion through normal pulmonary capillaries. Our methodology cannot, as yet, answer the question as to why MSCs are preferentially retained in pulmonary vasculature after infection. We hypothesize that a damaged tissue environment during pneumonia may result in leucocyte sequestration and MSC persistence. However, there is also evidence of MSC adhesion in vitro during inflamed conditions in our microfluidics model. Our studies indicate that this adhesion occurs after a period of physical entrapment, and is ICAM independent. The adhesive mechanisms underlying MSC sequestration and migration into inflamed lungs are still not well understood. Despite the high levels of constitutively expressed ICAM-1 within pulmonary vascular endothelial cells, the robust TNF- alpha-triggered MSC entrapment inside this model of lung vasculature was, in our hands, independent of ICAM.

Questions remain as to whether the efficacy of the MSCs is altered due to this retention as release of microvesicles has been shown to be a large part of their mechanism of action^4^. The majority of cell kinetic studies do not address whether the efficacy of MSCs is retained post administration. This is essential as consideration needs to be given to cell behaviour after ex-vivo environmental changes (such as labelling) prior to administration. The alteration of MSCs may indeed change their in vivo behaviour (removing surface markers, altering metabolism, proliferation and potency)^48,49^ leading to an altered response in vivo. Here, we assessed the efficacy of these tracked cells at attenuating *E. coli* pneumonia in our mouse model. We replicated previously observed outcomes whereby the indices of infection and physiological impairment were alleviated with the administration of unaltered MSCs^50,51^.

Microvesicles (MVs) are a potential therapeutic mechanism by which MSCs exert effects locally in pulmonary vasculature. MSCs have been shown to exert their beneficial effects via both paracrine and autocrine mechanisms. In recent years, there has been increasing interest in the potential use of MSC-derived extracellular vesicles as a cell-free alternative to whole cell MSC therapy. MVs are typically < 1 µM in size and are comprised of various proteins, lipids, mRNA and microRNA similar to the parent cell^52^. In particular, MVs released by MSCs have elicited therapeutic effects in preclinical^52,53^ and ex vivo human lung models of ARDS^54^. Previous work published by our group describes the therapeutic efficacy of MVs in a rodent model of pneumonia via monocyte phagocytosis and bacterial killing in particular. Elsewhere, the beneficial effects of MSC-derived MVs were decreased partially by pre-treatment of the MSCs with keratinocyte growth factor (KGF) siRNA^52^. This suggests that MVs do indeed contain growth factors which are central to the resolution of lung damage.

Finally, there is the potential for physical obstruction of the pulmonary vasculature with MSCs, and the effect of higher dosing strategies could exacerbate this. For example, early preclinical data has suggested that intracoronary delivery of MSCs is associated with safety issues, specifically microinfarction^55^ and ischemic ECG changes during and up to 7 days after cell delivery^56^. Recent studies have hinted at cell surface characteristics of MSCs that may promote microvascular thrombosis and pulmonary embolism after intravenous administration^57^.

However, the START trial by Matthay and colleagues used 1 × 10^7^ cells/kg, a dose they termed ‘high’, and found no hemodynamic or respiratory adverse effects related to administration of the cells to patients with moderate to severe ARDS^58^. Indeed, a trial conducted by Athersys used 900 million cells per patient (1.2–1.3 × 10^7^/kg) to test safety in ARDS which resulted in lower mortality and higher ICU-free days than controls and have also reportedly used 1200 million cells per patient (approximately 1.7 × 10^7^cell/kg) to treat ischemic stroke which has progressed to phase 3 trials.

The ideal dose of MSCs for differing severity of ARDS remains to be determined. In the current study, the dose of 5 × 10^5^ translates to approximately 2 × 10^7^ MSCs/kg. As this is at the upper limit of what has been investigated preclinically and clinically, albeit without adverse effects, an even higher dose would not be expected to have additional beneficial effects. Although caution is warranted and close monitoring of patients is needed, the clinical relevance of the obstructive and procoagulant effects of MSCs in humans is not certain considering that MSCs of a variety of sources have been proven safe in patients and thromboembolic events have not been reported^59^.

In summary, these findings confirm in real time the immediate entrapping and retention of MSCs following intravascular administration and their absence from the pulmonary vasculature after 24 h in healthy animals. Further to these findings we have demonstrated an MSC behaviour distinct from circulating leukocytes with no rolling or adhesion, and prolonged retention (> 24 h) in the vasculature of pneumonia models. The ultimate fragmentation of cells into microparticles gives insight into the paracrine functionality of MSCs often demonstrated in peripheral organ damage/disease after intravascular administration. In conclusion this study gives insight to the kinetics of MSCs administered to healthy and infected models and demonstrates the mechanism of clearance from the vasculature.

## Materials and methods

### Cell culture

Murine bone marrow mesenchymal stem cells (mMSCs) were received from Tulane University (Texas A&M: The Texas A&M Health Science Center College of Medicine Institute for Regenerative Medicine, TX, USA) as passage 7 cultures. mMSCs were expanded using Iscove’s Modified Dulbecco’s medium (IMDM, Sigma) containing 10% FCS (Atlanta Biologicals), 10% Horse serum (Hyclone), 100 U/mL penicillin, 100 µg/mL Streptomycin (Invitrogen), 0.25 µg/mL Amphotericin B and 2 mM L-Glutamine (Invitrogen) up to passage 12.

Human bone marrow mesenchymal stem cells (hMSCs) were received from Tulane University (Dr Prokop laboratory) as passage 2 cultures. hMSCs were expanded using minimum essential media-α (MEM-α, Glutamax, Gibco 32,561-029) containing 10% FCS (Atlanta Biologicals), and 100U/mL penicillin, 100 µg/mL Streptomycin (Invitrogen) and used between passages 3–5.

Human pulmonary microvascular endothelial cells (hPMVEC) were purchased from Lonza at passage 3 and expanded using Endothelial Cell Growth Medium supplemented with the EGM-2MV BulletKitT (Lonza) up to passage 12.

A Human leukemic monocyte lymphoma cell line (U937), was received as a kind gift from Dr Lisa Robinson (SickKids Research Institute, Toronto) and maintained in suspension culture using RPMI-1640 containing 10% FBS (Sigma).

All cells were grown under normal cell culture conditions at 37 °C, in a humidified atmosphere of 5% CO_2_, 21% O_2_.

### Mesenchymal stromal cell conditioned media (MSC-CM)

Human MSCs were cultured to 70% confluence in T175 flasks at passages 3–4 and then activated with either TNF-α (20 ng/mL) or LPS (50 ng/mL) overnight. Following activation, media was removed, cells were washed using 10 mL serum free MEM-α and incubated for a further 24 h with 15 mL serum-free α-MEM. Conditioned media was collected, and any non- adherent cells removed by centrifugation.

### Adhesion assays

To demonstrate static cell adhesion-potential of MSCs to the endothelium in vitro, 96 well plates were seeded with confluent monolayers of hPMVECs and treated with 20 ng/mL TNF- α for 4 h prior to the experiment as previously described^60,61^. MSCs were pre-treated with TNF-α (20 ng/mL for 1 h) or LPS (50 ng/mL for 24 h) prior to commencement of the adhesion assay. CFSE fluorescently labelled hMSCs (naïve or pre-treated) or U937 monocytes (naïve or exposed to MSC-CM from TNF-α or LPS pre-treated MSCs) were added to the wells and allowed to adhere for 1 h. Non-adherent cells were removed; the endothelial monolayers were washed with PBS and adherent cells quantified in each well by measuring fluorescence intensity and cell number calculated using standard curves for each cell type. In some experiments an anti-ICAM antibody (Clone: 20ug/ml) was preincubated with hPMVECs for 2 h prior to addition of the MSCs.

### Microfluidics

For the initial examination of cell adhesion under shear flow, confluent monolayers of hPMVECs were seeded in collagen coated chambers of Bioflux 48 well low shear plates (Fluxion Biosciences Inc.) and incubated for 24 h. Following incubation cells were activated by 20 ng/mL TNF-α for 4 h, the endothelial monolayer was washed once using fresh medium, and fluorescently labelled hMSCs and/or U937 monocytes (as endothelial binding capacity control) were applied at a shear stress of 2 dyne/cm^2^ which is a physiological flow rate relevant to leukocyte recruitment in postcapillary venules or the low shear environments in the pulmonary circulation^62^ using the BioFlux 200 system (Fluxion Biosciences Inc.). MSCs were observed for any interaction with the endothelium. In a subset of experiments the monolayer was exposed to 50 ng/mL LPS for 30 min to mimic endotoxin injury before the addition of hMSC cell suspensions. Cell adhesion was recorded continuously by a microscope camera via a Nikon Eclipse Ti inverted microscope and analysed by NIS-Elements BR software (Nikon Corporation, Japan).

### Animals

This study was carried out in compliance with the ARRIVE guidelines. 8 to 12-week-old C57BL/6 mice were purchased from Charles River Laboratories (Charles River Laboratories International, Inc.). Experiments were performed in accordance with Canadian Council on Animal Care (CCAC) guidelines and protocols were approved and monitored by the St Michael’s Hospital animal care committee.

For *E. coli* induced pneumonia, animals were subjected to anaesthesia using Ketamine (Ketamidor, 100 mg/kg) and Xylazine (Xylapan, 5 mg/kg) intraperitoneally and then orotracheally intubated using 22G IV Catheter (BD, Canada) under direct vision. 1 × 10^8^ *E. coli* CFU were administered followed by 2 × 400 µL air boluses. This dose had been selected based on pilot experiments as adequate to induce a moderate infection with less than 10% mortality.

### MSC Efficacy in E. coli pneumonia

In one group of mice, 5 × 10^5^ mMSCs were labelled using CFSE, re-suspended in 250µL sterile PBS, and administered via tail vein injection using a 30G needle 4 h after *E. coli* administration, control animals received IV vehicle. After another 44 h animals were surgically intubated using a 20G ET tube and sacrificed by exsanguination. Arterial blood was collected in sodium citrate collection tubes and plasma isolated. Samples were snap frozen in liquid nitrogen (LN2) and stored at − 80 °C. Following sacrifice lungs were excised from mice and wet weight recorded. Bronchoalveolar lavage (BAL) was performed using sterile 0.9% NaCl saline solution and collected for further analysis. Lobes from the right lung were separated and homogenised in 500µL of sterile PBS. The left lobe was inflated using 1:1 PBS:OCT compound, snap frozen in isopentane and stored for histological analysis, or inflated using PFA for stereological analyses.

BAL fluid was analysed for *E. coli* colony forming units (CFUs) by spreading 50 µL on TSA plates and incubating over-night at 37 °C. Total white cell counts in BAL were determined using a hemocytometer. Differential cell counts were performed by centrifuging BAL samples in cytospin cartridges at 200 rpm in a Cytospin centrifuge (Thermo Scientific) for 4 min, differential staining was performed, and cells were quantified. Lung tissue was analysed for CFU content by performing serial dilutions of the homogenate and plating on TSA plates.

### Intravital microscopy

Surgical procedures for intravital microscopy were performed as previously described^30^. Briefly, mice were anesthetised by intraperitoneal injection of Ketamine/Xylazine (200/10 mg/kg) and anaesthetic depth confirmed using paw pinch withdrawal. Surgical tracheostomy and jugular vein cannulation were performed and animals were ventilated at 100 breaths/minute with end-inspiratory and end-expiratory pressures of 11 and 1 cmH2O, respectively (Animal Respirator Compact 4600 Series, TSE Systems, Bad Homburg, Germany) under constant Ketamine/Xylazine anaesthesia using a syringe pump (11 Plus syringe pumps, Harvard Apparatus). The skin and muscle over the right rib cage was removed, and a 7- to 10-mm circular window was excised between the third and fifth ribs. A transdiaphragmal catheter (Portex Fine Bore Polythene Tubing, 0.58 mm ID/0.96 mm OD; Smiths Medical International) was placed into the right intrapleural space and the window was covered with a glass coverslip (5 mm diameter, Thomas Scientific) attached to a transparent polyvinylidene membrane (New Kure Wrap, Kureha, Tokyo, Japan) and tightly sealed with UltraGel Control glue (LePage, Henkle Corporations, CT, USA). Intrathoracic air was removed via the intrapleural catheter, thus establishing direct coupling of the lung surface to the transparent window membrane. After completion of the surgical preparation, mice were transferred to an upright microscope (Axiotechvario 100HD; Zeiss, Jena, Germany) on a custom-built, computer-controlled stage. After a 30-min stabilization period, fluorescently labelled MSCs (5 × 10^5^ cells) were administered in a 300µL bolus via the intrajugular catheter. Fluorescence was excited at 495 nm by monochromatic illumination (Polychrome IV; T.I.L.L. Photonics, Martinsried, Germany), collected through appropriate objectives (Achroplan 10x/0.30 W, Achroplan 20x/0.50 W, Achroplan 40x/1.2 W; Zeiss) and dichroic and emission filters (FT 510, LP520; Zeiss), imaged by a silicone intensified tube camera (CF 8/4 FMC; Kappa, Gleichen, Germany) and recorded on digital videotape (DVCAM, DSR-25, Sony Deutschland, Berlin) at 0, 60 and 120 min following MSC administration. At the end of the protocol, the plasma marker FITC dextran (0.2 mL of a 1% solution; molecular mass 150 kDa, Sigma Chemical, St. Louis, MO) was infused intravenously to allow for the visualization of pulmonary arterioles, capillaries, and venules on the surface of the right upper lung lobe. In some experiments, Rhodamine 6G (100 μg/mL, Sigma) for in vivo staining of circulating leukocytes was injected via the venous catheter and imaged as described.

Confocal intravital microscopy was performed on the animals immediately post-mortem at different time-points. Z-stacked images of intravascularly lodged fluorescent MSCs were compiled using ImageJ software to demonstrate the 3D structure of the cells and their shape and fragmentation in vivo.

## Supplementary Information

Supplementary Video 1.

Supplementary Video 2.

Supplementary Video 3.

Supplementary Video 4.

Supplementary Video 5.

Supplementary Video 6.

Supplementary Figures.
