# Supplementary material for: Intra-vital imaging of mesenchymal stromal cell kinetics in the pulmonary vasculature during infection
Source: Sci Rep. 2021 Mar 4;11:5265. doi: 10.1038/s41598-021-83894-7 (PMC7933415; doi:10.1038/s41598-021-83894-7)
Supplement: Supplementary file 7 — Supplementary Figures. [file 41598_2021_83894_MOESM7_ESM.docx]

**Supplemental Figure 1**

2500

MSC binding to hPMVEC (Fluorescence@488nm)

2000

1500

1000

500

0

hPMVEC ICAM1

antibody

DAPI

**2.**


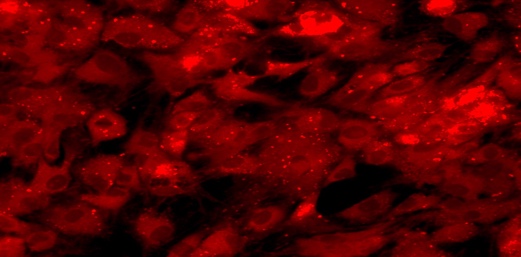
hPMMEC’s– CellTracker™ Red


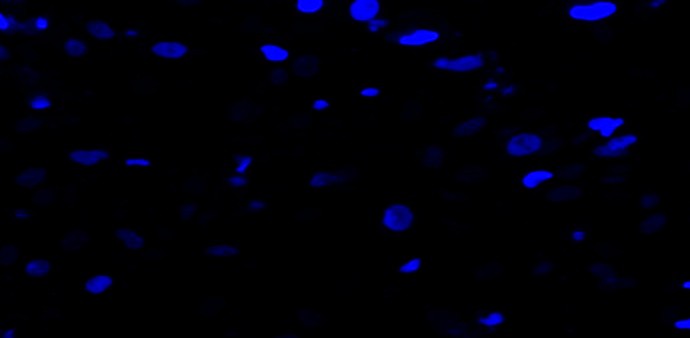


**1.**


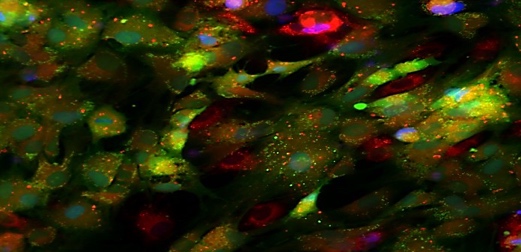
**4.**


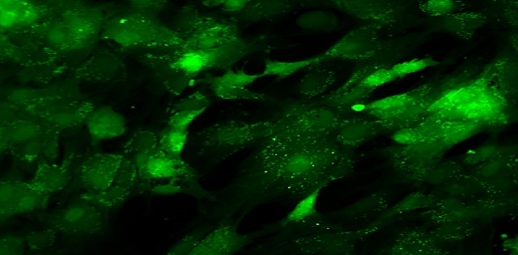


**3.**

MSC’s – Calcein Green

Merged

**Supplemental Figure 1:** *In situ* adhesion assays were performed using fluorescently labelled MSCs added to TNF-α-activated monolayers of fluorescently labelled hPMVECs. An anti-ICAM antibody preincubated with hPMVECs for 2 hrs prior to addition of the MSCs did not affect MSC binding to hPMVEC. (hPMVEC = human pulmonary microvascular endothelial cells).

**Supplemental Figure 2**


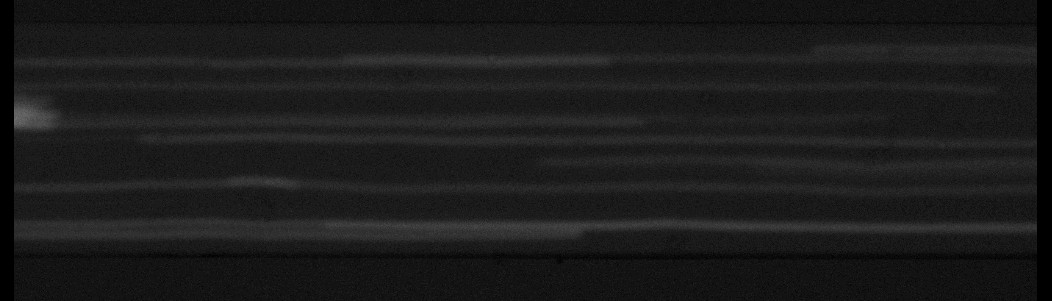
A B


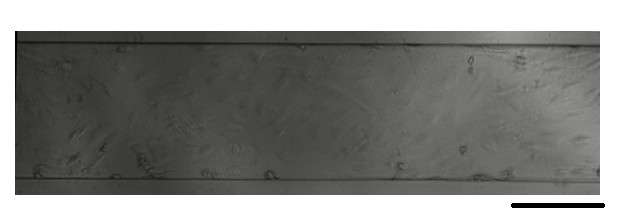


100µm

Non-Primed MSCs


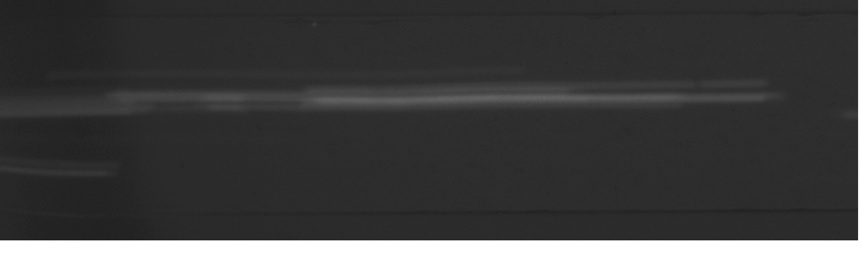
C D


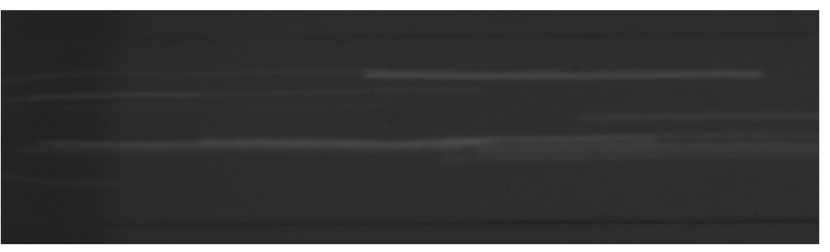
TNFα-Primed MSCs

LPS-Primed MSCs

**Supplemental Figure 2:** Bioflux microfluidics channels were seeded with monolayers of hPMVECs allowed to adhere overnight followed by 4h of TNF-α stimulation (A). The application of 2dyn/cm2 pressure allowed fluorescently labelled MSCs to pass over the monolayer under shear flow. Still images from active flow experiments demonstrated no rolling or adhesion of either Naïve MSCs (B), TNF-α activated MSCs (C) or LPS activated MSCs (D). TNF-α and LPS activated MSCs tended to aggregate in the system. MSC: Mesenchymal Stem Cell; LPS: Lipopolysaccharide; Mo: Monocyte; hPMVEC: human pulmonary microvascular endothelial cells.


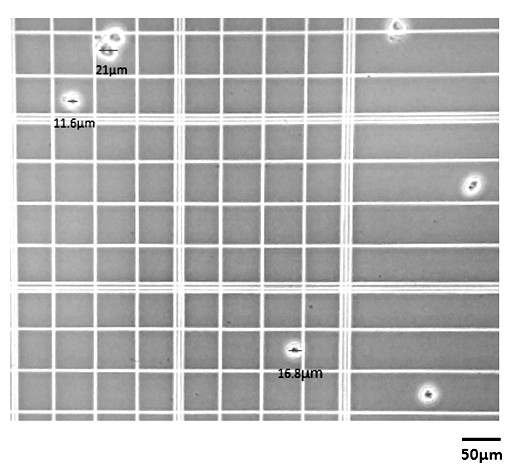
**Supplemental Figure 3**


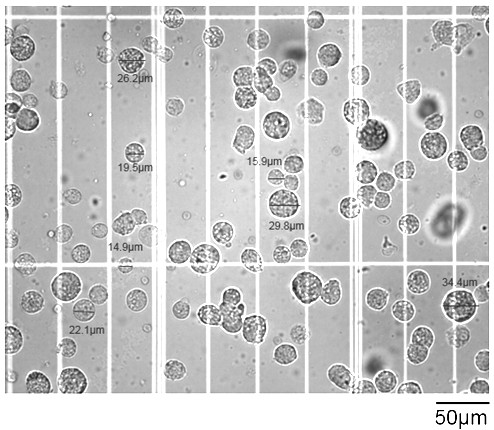
A B


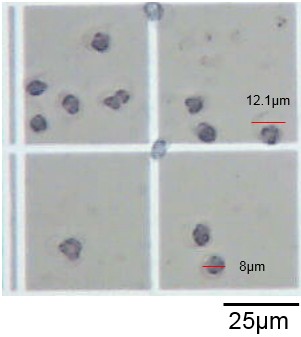
**40**

C D

**Cell Diameter (µm)**

**30**

**20**

**10**

**0**

**hMSC**

**mMSC**

**mWBC**

**Supplemental Figure 3:** Different cells of interest were measured using a haemocytometer *ex vivo*. A comparison of the diameters of hMSCs (A), mMSCs (B), and BAL WBCs (C) demonstrate a marked size difference between the cells and a range of cell sizes in the hMSC sample (D). MSC: Mesenchymal Stem Cell; BAL: Bronchioalveolar lavage; WBC: White Blood Cell.

## Supplemental Figure 4


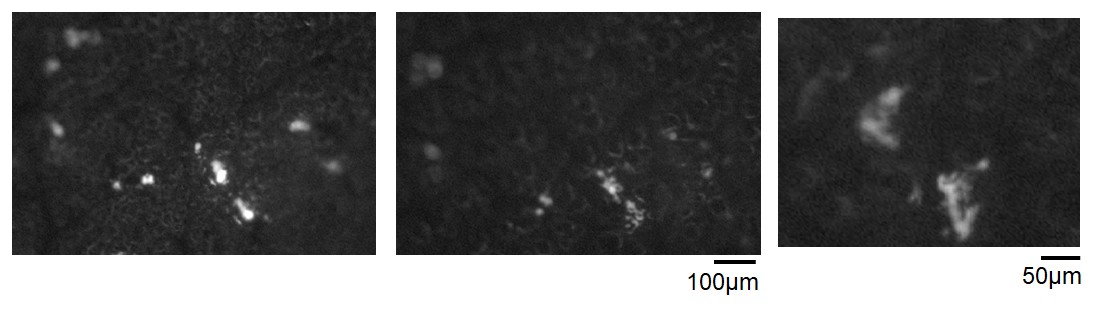


**Supplemental Figure 4:** Observation of MSCs over time during IVM revealed a significant change in cell morphology within the vessels. MSCs at 0min post cell administration exhibited a rounded, regular shape (i), whereas the same MSCs at 120min post administration demonstrated a distorted morphology (ii) which was more evident at higher magnifications (iii). The movement of the lung and heart during IVM prevented closer analyses of these cells.

MSC: Mesenchymal Stem Cell; IVM: Intravital Microscopy.

0 min (10X)

120 min (10X)

120 min (40X)

i

ii

iii

**Supplemental Figure 5**


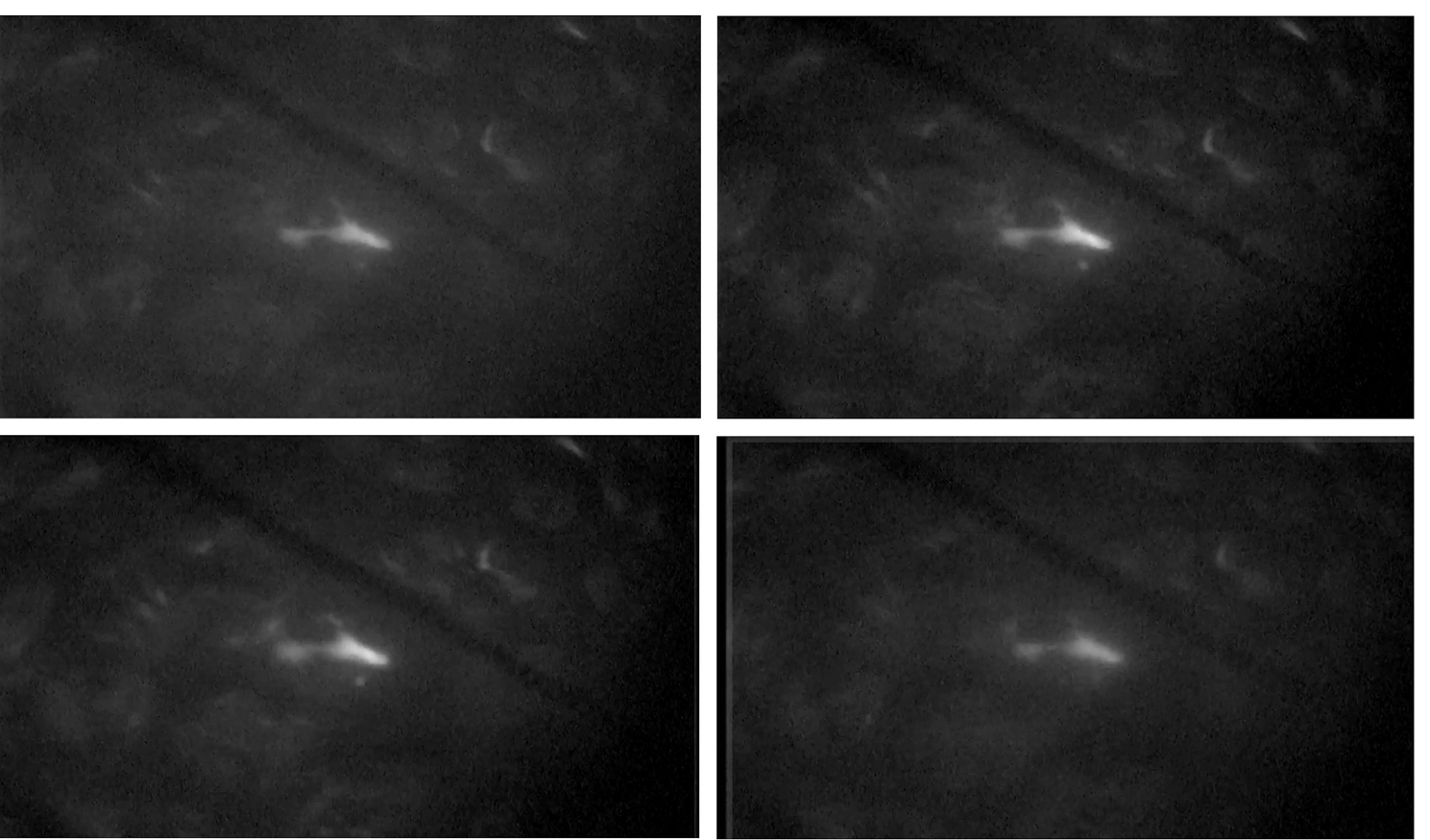


i

ii

iii

iv

**Supplemental Figure 5:** The use of high magnifications during IVM allowed the visualisation of the emergence and release of cell particles approximately 40-60 minutes post cell administration. The release of these particles was tracked and the particle visible 60 min post administration (i) moved as if still attached to the cell or vasculature (ii, iii) and was eventually released (iv).

MSC: Mesenchymal Stem Cell; IVM: Intravital Microscopy.
